# Performance of Streck cfDNA Blood Collection Tubes for Liquid Biopsy Testing

**DOI:** 10.1371/journal.pone.0166354

**Published:** 2016-11-10

**Authors:** Inga Medina Diaz, Annette Nocon, Daniel H. Mehnert, Johannes Fredebohm, Frank Diehl, Frank Holtrup

**Affiliations:** Research and Development, Sysmex Inostics GmbH, Hamburg, Germany; Hospital Authority, CHINA

## Abstract

**Objectives:**

Making liquid biopsy testing widely available requires a concept to ship whole blood at ambient temperatures while retaining the integrity of the cell-free DNA (cfDNA) population and stability of blood cells to prevent dilution of circulating tumor DNA (ctDNA) with wild-type genomic DNA. The cell- and DNA-stabilizing properties of Streck Cell-Free DNA BCT blood collection tubes (cfDNA BCTs) were evaluated to determine if they can be utilized in combination with highly sensitive mutation detection technologies.

**Methods:**

Venous blood from healthy donors or patients with advanced colorectal cancer (CRC) was collected in cfDNA BCTs and standard K_2_EDTA tubes. Tubes were stored at different temperatures for various times before plasma preparation and DNA extraction. The isolated cfDNA was analyzed for overall DNA yield of short and long DNA fragments using qPCR as well as for mutational changes using BEAMing and Plasma Safe-Sequencing (Safe-SeqS).

**Results:**

Collection of whole blood from healthy individuals in cfDNA BCTs and storage for up to 5 days at room temperature did not affect the DNA yield and mutation background levels (n = 60). Low-frequency mutant DNA spiked into normal blood samples as well as mutant circulating tumor DNA in blood samples from CRC patients collected in cfDNA BCTs were reliably detected after 3 days of storage at room temperature. However, blood samples stored at ≤ 10°C and at 40°C for an extended period of time showed elevated normal genomic DNA levels and an abnormally large cellular plasma interface as well as lower plasma volumes.

**Conclusion:**

Whole blood shipped in cfDNA BCTs over several days can be used for downstream liquid biopsy testing using BEAMing and Safe-SeqS. Since the shipping temperature is a critical factor, special care has to be taken to maintain a defined room temperature range to obtain reliable mutation testing results.

## Introduction

The accumulation of genetic and epigenetic alterations is responsible for the transformation of normal human cells into cancer cells. The detection of these DNA variations in tumor tissue is therefore important for a number of diagnostic applications. An alternative and minimally invasive source of tumor DNA is circulating cell-free DNA that can be isolated from plasma or serum, an approach described as liquid biopsy [1]. Various applications of circulating tumor DNA analysis in clinical oncology research have been proposed with some of them now reaching routine clinical testing [2,3]. All these applications require highly sensitive procedures like digital PCR or barcoded next-generation sequencing approaches to detect the minuscule amounts of ctDNA among a majority of wild-type cfDNA [4].

Comparable to other *in vitro* diagnostic tests, the influence of pre-analytical steps on the overall test performance has to be understood and well defined since harmonized standards are not yet available [3,5–8]. For the analysis of cfDNA, these steps include blood collection comprised of the blood draw itself, as well as the handling, shipment and storage of the tube, followed by the plasma processing as well as DNA extraction. One of the major factors that influence the sensitivity of detecting cfDNA within this workflow is the unintended release of wild-type DNA due to white blood cell (WBC) lysis between the time of blood draw and processing of plasma. The risk of an increased wild-type genomic DNA amount may impair the detection of rare ctDNA molecules if it were to exceed the analytical selectivity of the downstream ctDNA detection method. More specifically, mutant allele frequencies (MAFs) across a variety of cancer types and stages can be as low as one mutant DNA copy per 10,000 normal wild-type DNA molecules [9,10]. Thus, any unintended increase in the concentration of normal DNA in the plasma sample might adversely affect the detectability of mutant ctDNA even though the detection technology is highly sensitive. Enabling the preparation of cfDNA without random release of normal genomic DNA from WBCs is therefore an important and critical pre-analytical factor. One way to achieve this is to process blood samples immediately after collection and perform additional high-speed centrifugation steps during subsequent plasma processing [6,11,12]. In clinical practice, this is not always possible because blood collection sites are usually not equipped to prepare plasma by high-speed centrifugation and to subsequently ship frozen plasma to a testing laboratory. An alternative approach to maintain the cfDNA population is to inhibit nuclease activity and stabilize WBCs in the blood collection tube itself. For instance, Cell-Free DNA BCT tubes (cfDNA BCTs) commercialized by Streck (La Vista, NE) are designed to provide these sample stability features for up to 14 days at temperatures between 6°C and 37°C [13]. These tubes have already been widely applied for non-invasive prenatal testing (NIPT) where the average fetal fraction in the maternal plasma is 10–15% when measured between gestational weeks 10 and 20 [14]. In this MAF range, any unintended release of genomic DNA from lysed WBCs is less critical. However, in oncological samples where the abundance of ctDNA in the cfDNA population can be as low as 0.01% and quantification of the allele frequency can add clinically critical information. Therefore, for any clinical application where high sensitivity is necessary any factor that may increase the wild-type DNA fraction is not desired. In consequence, more stringent usability studies have been performed to assess the feasibility of cfDNA BCTs for oncological applications [5,11,15]. These studies have already shown promising results. However, several open questions need to be answered so as to engender additional confidence in the routine introduction of alternative blood collection tubes in oncology. First, do cfDNA BCTs allow the stabilization of cfDNA and prevent release of additional wild-type DNA from blood cells over the time period of a typical shipment process? Second, does the cell stabilizer affect the downstream ability to amplify the cfDNA? Third, does the cell stabilizing agent induce DNA damage resulting in false positive mutation results or does it affect the detectability of rare mutations? And fourth, what happens to the blood sample and subsequent analysis if storage and transportation conditions deviate from room temperature? To answer these questions, we evaluated whole blood stored in cfDNA BCTs and standard K_2_EDTA blood collection tubes side-by-side by real-time quantitative PCR as well as the highly sensitive BEAMing and Plasma Safe-Sequencing technologies [10,16]. We employed these techniques to investigate blood samples from healthy volunteers with and without artificial mutant DNA spike-ins as well as from patients with colorectal tumors.

## Materials and Methods

### Ethics statement

Ethics committee approval was not required for this specific work as the samples were collected commercially from BIOMEX GmbH (Heidelberg, Germany) and Indivumed GmbH (Hamburg, Germany), respectively. BIOMEX GmbH has obtained institutional review board approval for the collection of the samples by the Bavarian Chamber of Physicians Ethics Committee (05142), and Indivumed GmbH has obtained approval from the institutional review board by the Physicians Association of Hamburg, Germany (PV2963). Written consent was obtained from all study subjects by sample providers. All clinical data and samples were received by Sysmex Inostics GmbH anonymously.

### Blood collection

**Fig 1:**
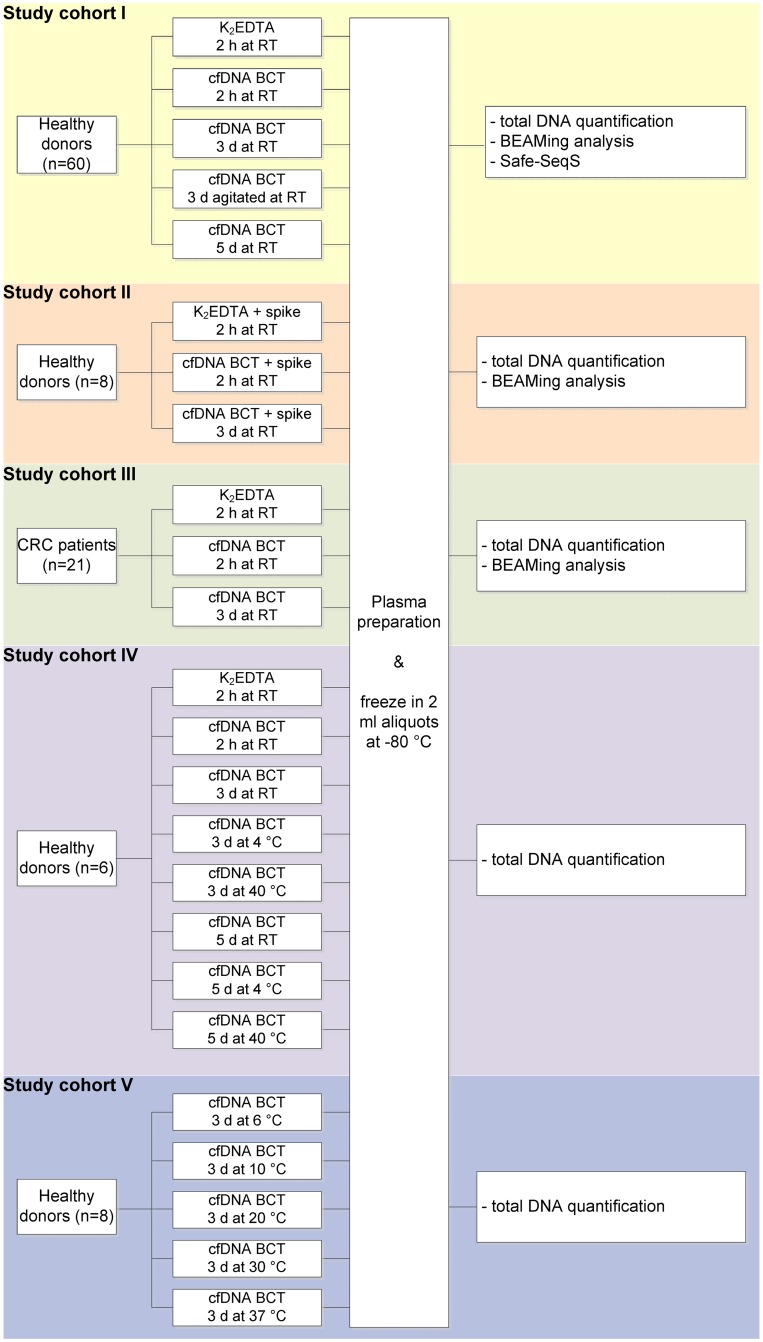
Experimental study cohorts. Experimental setup for cfDNA BCT vs K_2_EDTA performance experiments. Cohort I: Time point experiments at room temperature including DNA quantification and mutation analysis using BEAMing and Safe-SeqS. Cohort II: BEAMing analysis of blood samples spiked with synthetic double-stranded mutant DNA fragments at different allele frequencies. Cohort III: BEAMing analysis of samples collected from colorectal cancer (CRC) patients. Cohort IV: Experiment evaluating effects of extreme storage temperatures on DNA quantity. Cohort V: Experimental evaluation of recommended temperature range.

Venous blood from presumably healthy donors and patients with colorectal cancer (stage II-IV) was collected by standard phlebotomy techniques in BD Vacutainer K_2_EDTA tubes (Becton Dickinson, Franklin Lakes, NJ; referred to as K_2_EDTA tubes) and Cell-Free DNA BCT tubes (Streck, La Vista, NE; referred to as cfDNA BCTs). All tubes were filled to 10 ml as recommended by both manufacturers. After the blood draw, tubes were inverted 10 times and transported at room temperature (RT) to the laboratory for processing and storage according to the scheme in Fig 1. To simulate movement during a potential shipping process, a subset of cfDNA BCTs was constantly agitated for 3 days.

### Simulation of blood samples containing mutant DNA

To simulate blood samples with defined MAFs, synthetic double-stranded DNA fragments (gBlocks, Integrated DNA Technologies, Coralville, IA) harboring specific point mutations were prepared in a background of human genomic DNA (Promega, Madison, WI) and injected into whole blood samples. To mimic the size distribution of cfDNA, 85 μg human genomic DNA (Promega) were fragmented to a median size of 160 base pairs (bp) by a NEBNext dsDNA Fragmentase (New England Biolabs, Ipswich, MA) for 30 min at 37°C according to the manufacturer’s instructions. Following an EDTA inactivation step, fragmented DNA was immediately purified using a MinElute PCR purification kit (Qiagen, Hilden, Germany) following the manufacturer’s instructions. The DNA size distribution of fragmented samples was analyzed utilizing a 2100 Bioanalyzer (Agilent Technologies, Santa Clara, CA); yields of DNA were quantified applying the 96 bp *long interspersed nuclear elements* (LINE-1) qPCR assay, as described below. Mutant gBlock DNA fragments mimicking the size of ctDNA (200 bp for *PIK3CA* c.3140A>G, 140 bp for *EGFR* c.2369C>T and 192 bp for *KRAS* c.34G>A) were mixed with fragmented genomic DNA to create the spike mixes for incorporation into the whole blood samples. Using insulin syringes, 200 μl of the spike mixes containing the *PIK3CA* (0.1% MAF; 60 copies), *EGFR* (0.5% MAF; 300 copies) and *KRAS* (1% MAF; 600 copies) variants in a background of 60,000 genome equivalents (GEs) of fragmented DNA were injected into the blood samples immediately after the blood draw.

### Plasma preparation

After indicated storage times (see Fig 1), blood tubes were centrifuged at 1,600 × g for 10 min at RT using a swing-out rotor. A smooth braking profile was used to prevent disruption of the buffy coat layer. Collection tubes were then carefully removed to avoid turbulences of the buffy coat. Using a 1 ml pipet, plasma was transferred to a fresh 15 ml tube leaving approximately 500 μl plasma in the blood collection tube to avoid transfer of the cellular fraction. In samples with almost no visible clear plasma, at least 2.5 ml supernatant was transferred from the top (occurred in extreme storage temperature conditions, see cohort IV in Fig 1). To remove any residual blood cells, the supernatant was centrifuged a second time at 6,000 × g for 10 min at RT using a swing-out rotor and a smooth breaking profile. The supernatant was transferred again to a fresh tube leaving approximately 300 μl above the potential pellet to avoid transfer of any cellular debris. Plasma was gently mixed by pipetting, aliquoted in cryogenic vials, and frozen at -80°C until DNA extraction. Plasma preparation was carried out without break points between individual steps.

### DNA extraction

2 ml plasma aliquots were thawed and immediately processed using the QIAamp Circulating Nucleic Acid Kit (Qiagen). The manufacturer’s instructions were followed except for the proteinase K incubation time at 60°C, which was extended from 30 to 60 min as recommended by Streck for plasma collected with cfDNA BCTs. The extended incubation times were also applied to the K_2_EDTA samples to ensure comparability. The DNA was eluted in 140 μl AVE buffer and stored at 4°C until further analysis.

### DNA quantification by LINE-1 qPCR

DNA was quantified by a qPCR assay specific for the LINE-1 sequence as described earlier [17]. A short 96 bp LINE-1 amplicon was designed to allow effective quantification of cfDNA which is typically smaller than 180 bp [18]. In contrast, a 402 bp amplicon was utilized to preferentially quantify genomic DNA released from WBCs [19]. The 96 bp amplicon was selected from within the sequence of the 402 bp amplicon. The 402:96 bp ratio served as an indicator for the quality of a cfDNA sample. High values obtained with this size determination assay indicate dilution of cfDNA with wild-type DNA released from blood cells during storage or plasma preparation, whereas low values indicate uncompromised cfDNA quality. For the qPCR assay, 2.5 μl extracted DNA were diluted 1:10 in TE buffer pH 8.0, and 5 μl of the diluted DNA sample were combined with 5 μl LINE-1 master mix (Sysmex Inostics GmbH, Hamburg, Germany) for quantification on an Applied Biosystems 7500 Fast Dx thermocycler (Thermo Fisher Scientific, Waltham, MA). Cycling conditions were 95°C for 2 min, then 95°C for 15 s, 59°C for 15 s and 70°C for 30 s cycled 35 times. Human genomic DNA (Promega) serially diluted from 303 to 0.3 GE/μl (1 GE = 3.3 pg) was used as a reference standard. All reactions were run in triplicate.

### Mutation analysis in cfDNA

Mutation analysis was performed using BEAMing [20] and Safe-SeqS [16]. Depending on the study cohort, various mutation panels were applied to test for common alterations in *KRAS*, *PIK3CA*, or *EGFR* genes. In the BEAMing procedure, targeted DNA sequences were amplified on magnetic beads during emulsion PCR and hybridized with wild-type or mutant sequence-specific fluorescent probes. Discrimination of wild-type vs mutant beads was performed using a C6 flow cytometer (Becton Dickinson) and analyzed by FCS Express 4.0 (De Novo Software, Glendale, CA). All samples exhibiting a mutant bead population of greater than 0.02% of all beads carrying the target PCR product were considered tentative mutant positive. This value was therefore used as the cut-off for the KRAS BEAMing mutation panel. Tentative mutant positive results were further checked for plausibility by calculating the number of mutant DNA molecules per sample (mutant fraction multiplied by the input GEs). Samples with at least one mutant molecule were determined to be true mutants.

A Safe-SeqS panel covering five *c-KIT* amplicons was applied to gain per-base resolution on the mutation background in wild-type samples. Briefly, cfDNA molecules were uniquely labeled with a random DNA identifier (UID) during an initial PCR step. After UID assignment, a second amplification was performed using well-specific barcodes. Following a purification step, the PCR products were redundantly sequenced on a MiSeq (Illumina, San Diego, CA) with a distinct UID coverage of >30.000-fold on average. Data analysis was performed by deconvolution of the reads into UID families, with a minimum of 4 sequencing reads sharing a common UID sequence. The presence of a specific common mutation in at least 90% of UID family members qualified the mutation as a true positive. Single nucleotide polymorphisms (SNPs) reported in dbSNP (http://www.ncbi.nlm.nih.gov/SNP) were removed from the analysis, and no additional filtering steps were applied.

### Statistical analysis

Statistical analysis using one-way ANOVA and linear regression with R^2^ calculation were performed in GraphPad Prism 6.07 (GraphPad Software, Inc., La Jolla). P-values of p<0.05 were deemed significant. Graphs were generated in GraphPad Prism.

## Results

### cfDNA BCTs stabilize cfDNA and prevent genomic DNA release for at least 5 days at RT

Making liquid biopsy testing widely available requires a strategy to ship whole blood for several days while retaining cfDNA integrity and blood cell stability. It is known that WBCs start to lyse over time in the commonly used K_2_EDTA blood collection tubes; this leads to an elevated DNA background, thus diluting the circulating tumor DNA population [5]. To assess the WBC-stabilizing potential of cfDNA BCTs, blood was collected from 60 healthy donors and stored at RT for up to 5 days prior to plasma processing. Constant agitation of blood samples was used to mimic movement during the intended shipping time of 3 days (Fig 1, study cohort I). These timeframes were selected since it is assumed that the shipment of whole blood to a central testing laboratory typically is completed within 3 days [21]. A size determination assay was applied to evaluate DNA release from WBCs based on the calculation of the amount and ratio of two differently sized LINE-1 fragments (402:96 bp ratio).

**Fig 2:**
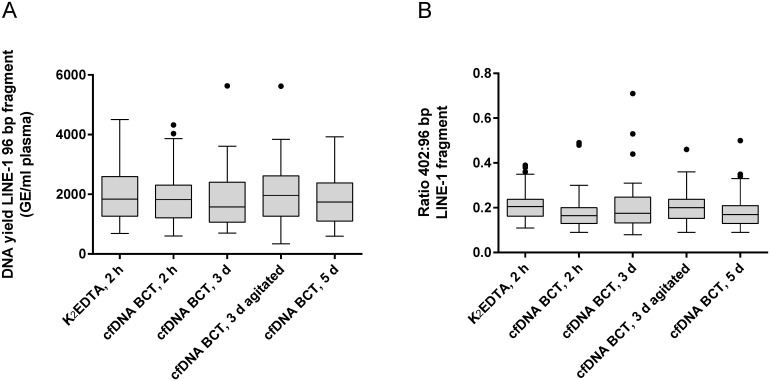
Analysis of cfDNA yield and genomic DNA release for study cohort I. (A) DNA yield was assessed for cfDNA from blood samples stored at room temperature (18°C– 22°C) in K_2_EDTA tubes vs cfDNA BCTs (healthy donors, n = 60). Plasma was prepared after indicated storage conditions. Extracted DNA was analyzed for overall yield by qPCR amplifying a 96 bp LINE-1 fragment. (B) Illustration of the DNA yield ratio between long (402 bp) and short (96 bp) LINE-1 fragments (n = 60). Increased ratios compared to K_2_EDTA reference would indicate genomic DNA release. Shown are box plots with 1.5 × interquartile range (IQR) applied to create whiskers and outliers. Statistical analysis using one-way ANOVA revealed no significant difference between conditions.

As shown in Fig 2, no significant difference was detected with respect to cfDNA quantity (long and short fragment) in the 60 healthy donor samples collected in K_2_EDTA vs cfDNA BCTs and processed directly after blood draw (K_2_EDTA and cfDNA BCTs) or after prolonged storage at RT for 3 and 5 days (cfDNA BCTs only). Constant agitation of blood in cfDNA BCTs for 3 days did not elevate the DNA amount indicating that cfDNA BCTs effectively prevent DNA release from WBCs under conditions suitable for whole blood shipping. In all conditions, the cfDNA quantity was within the expected range for healthy donors with a median of ~2000 GE/ml plasma as reported previously [22]. The stabilizing effect on WBCs and cfDNA integrity is also reflected by the results of the size determination assay which presents a median 402:96 bp ratio of ~0.2 for all conditions (Fig 2B), i.e. ~20% of the quantifiable DNA likely originates from WBCs [23]. The median plasma volume obtained from healthy donors (n = 60) was found to be almost identical for both tube types at the 2 hours RT condition with medians of 5.5 ml for K_2_EDTA tubes and 5.7 ml for cfDNA BCTs (S1 Fig). Across the prolonged storage period a slight decrease of obtained plasma volumes was found (S1 Fig).

### Wild-type samples stored in cfDNA BCTs are PCR-amplifiable and do not show increased mutation rates

Many cell stabilizers contain cross-linking reagents which may induce DNA damage and lead to an increased mutation profile in wild-type DNA as well as interfere with PCR amplification [24,25]. To assess the amplifability of cfDNA obtained from BCTs, a standard endpoint PCR reaction was performed on the samples from study cohort I (Fig 1) and the PCR products were subsequently accessed by agarose gel electrophoresis. No visible difference in band intensity between the tested conditions was observed (data not shown). This demonstrates unimpaired PCR accessibility of cfDNA exposed to the cfDNA BCT stabilizer for up to 5 days.

**Fig 3:**
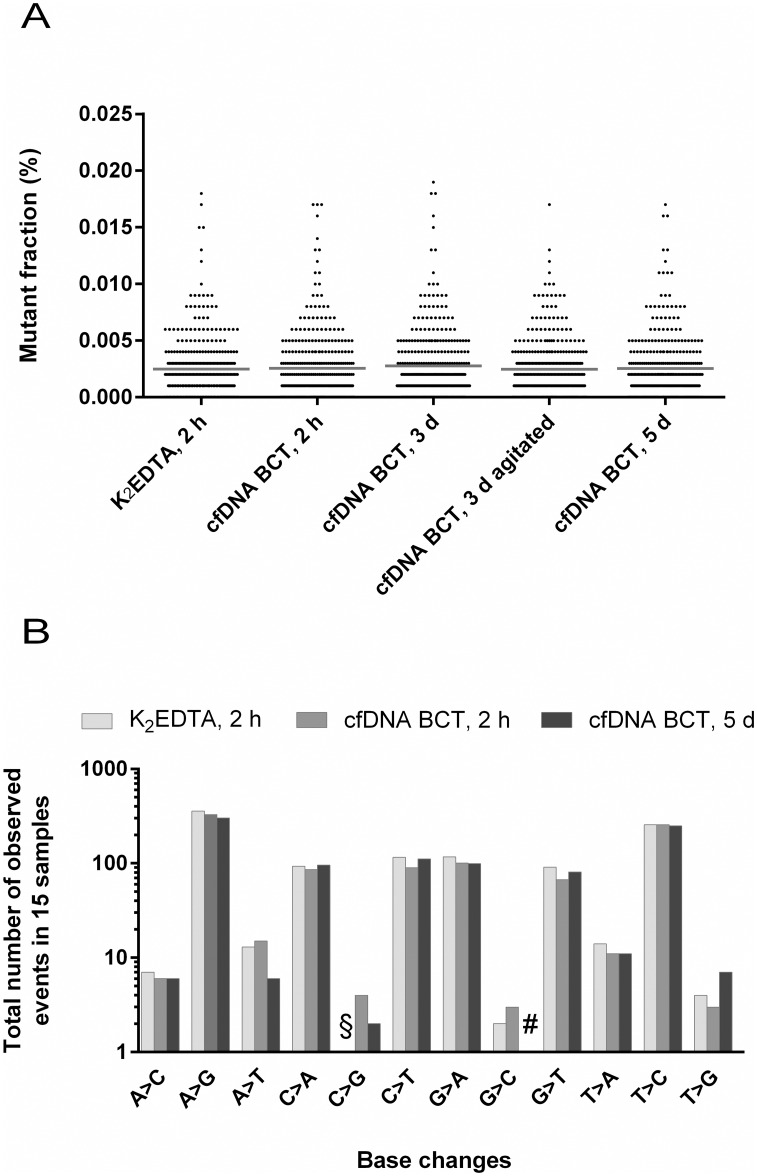
Mutation background analysis of study cohort I using BEAMing and Safe-SeqS. (A) Mutation background was assessed using BEAMing for 6 *KRAS* mutations (c.34G>A, c.34G>C, c.34G>T, c.35G>A, c.183A>T, c.437C>T) in presumably healthy wild-type donors (n = 60). Samples were stored at room temperature. Shown are scatter dot plots with mean values (horizontal line). One-way ANOVA statistics revealed no statistical significance between conditions. (B) Safe-SeqS was applied to analyze base changes in five *c-KIT* amplicons from healthy donor blood samples stored in K_2_EDTA tubes and cfDNA BCTs at RT for the indicated time (n = 15). In total, 553 bases were analyzed per sample (171 As (31%), 105 Cs (19%), 110 Gs (20%) and 167 Ts (30%)). §: 1 event detected for K_2_EDTA 2 h C>G; #: 0 events detected for cfDNA BCT 5 d G>C.

To assess if prolonged storage of blood from presumably healthy (i.e. wild-type) donors in BCTs leads to an elevated false positive mutation rate, all DNA samples from study cohort I were tested for 6 *KRAS* mutations by BEAMing. The resulting 360 mutant fraction data points for each test condition are illustrated in Fig 3A. None of the data points exceeded the predefined cut-off of 0.02% above which a measurement would be considered mutant. Additionally, the average background mutation rate was almost identical for all conditions. Furthermore, there was no significant difference in the distribution of the data points between the K_2_EDTA reference and all cfDNA BCT storage conditions indicating that the stabilizer does not increase the background in wild-type DNA analyzed by BEAMing.

To expand the data set obtained by BEAMing, K_2_EDTA, cfDNA BCT 2 h and cfDNA BCT 5 d conditions of 15 randomly selected donors were additionally tested for sequence variations in five *c-KIT* amplicons using Safe-SeqS. In total, 553 bases were interrogated in each sample and condition. The results from these experiments are shown in Fig 3B, where the number of detected base changes per condition cumulated for all 15 donors was analyzed. Compared to the K_2_EDTA reference, there was no increase of the background obtained for any of the possible base changes in samples stored in cfDNA BCTs. These results confirm that the stabilizer in cfDNA BCTs does not induce DNA damage if stored for up to 5 days.

### Storage of blood in cfDNA BCTs does not impair detectability of low-level mutations

**Fig 4:**
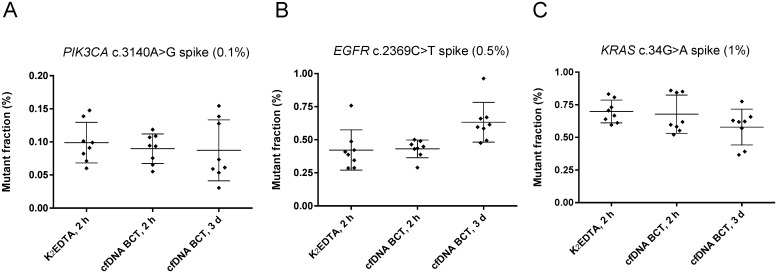
Detectability of low frequency mutations in study cohort II. Blood from healthy donors (n = 8) was spiked with synthetic double-stranded mutant DNA fragments of different allele frequencies in a background of 60,000 genome equivalents of fragmented human genomic wild-type DNA and subsequently stored at RT. DNA was extracted and analyzed by BEAMing after the indicated storage time. Mean and standard deviation of the detected mutant fraction is shown. (A) *PIK3CA* c.3140A>G spike (0.1%), (B) *EGFR* c.2369C>T spike (0.5%), (C) *KRAS* c.34G>A spike (1%).

Having demonstrated that the cfDNA BCT stabilizer does not increase the mutation background in wild-type samples, we examined samples collected in these tubes with respect to the ability to detect mutant DNA. Low-level mutant samples were chosen since MAFs in clinical specimens are frequently <1%, and detectability of such levels should not be impaired after fixation in cfDNA BCTs [26,27]. For this evaluation, samples with defined MAFs of 0.1% (*PIK3CA* 1340A>G), 0.5% (*EGFR* 2369C>T) and 1% (*KRAS* 34G>A) were prepared as outlined in the methods section (Fig 1, study cohort II). BEAMing was used to determine the frequency of the spiked mutations in these samples after the intended shipping time of 3 days vs 2 h controls (Fig 4).

No significant differences were found between the average mutant fraction detected in K_2_EDTA tubes and cfDNA BCTs. For the majority of measurements, the anticipated mutant fraction was found within the expected range of ± 50% of the target value. Compared to the K_2_EDTA storage conditions, samples stored in cfDNA BCTs for 3 d showed a slightly increased average mutant fraction for the *EGFR* spike (0.6% vs 0.4%, Fig 4B) and a slightly reduced fraction for the *KRAS* spike (0.6% vs 0.7%, Fig 4C), most likely caused by statistical variation. If each cfDNA BCT mutant fraction value is normalized to its corresponding K_2_EDTA reference value, the average fold difference is not changed and the spread of the normalized values lies within the ± 50% range (S2 Fig). Thus, our observations do not point towards an impairment of detectability of mutations after blood was stored in cfDNA BCTs.

### Blood samples from CRC patients collected in cfDNA BCTs vs K_2_EDTA tubes yield highly comparable mutation testing results

To verify the results obtained with wild-type and simulated mutant samples with clinically relevant specimens, blood from 21 colorectal cancer patients was collected in K_2_EDTA tubes and cfDNA BCTs (Fig 1, study cohort III). Plasma was prepared either after 2 h or after 3 d of storage at RT, and samples were subsequently tested for 33 different mutations in the *KRAS* and *NRAS* genes using the OncoBEAM RAS panel, a standardized BEAMing approach [28].

**Fig 5:**
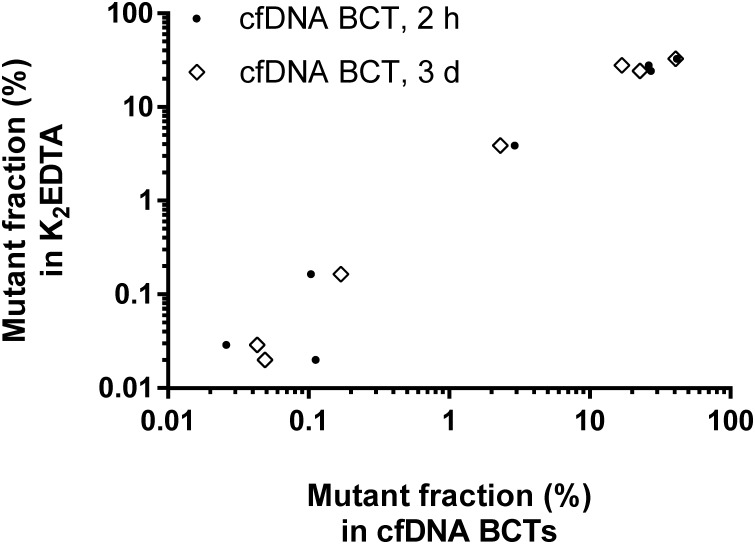
Correlation of mutation analysis results from CRC samples collected in K_2_EDTA and cfDNA BCTs (study cohort III). Blood from CRC patients (n = 21) was collected in K_2_EDTA tubes and cfDNA BCTs. Samples were processed after the indicated storage time and analyzed using the OncoBEAM RAS assay (33 *RAS* mutations). Mutant fractions for all *RAS*-positive events are plotted for both cfDNA BCT conditions vs matched K_2_EDTA reference (n = 7 per condition).

The DNA yield was found to be highly comparable between the different tube and storage conditions for the matching patient samples. A total of 7 mutations were detected in 6 out of 21 samples (28.6%) with one patient harboring a mutation in both the *KRAS* and *NRAS* genes (S1 Table). Mutant allele frequencies in the cfDNA ranged from 0.025% to 41.6%. A 100% concordance was obtained regarding the qualitative BEAMing test results (mutant or wild-type) in the K_2_EDTA condition and the corresponding cfDNA BCT conditions. The mutant fraction determined for the 7 identified mutations demonstrated a high correlation between the conditions (Fig 5): R^2^ = 0.973 for K_2_EDTA (2 h) vs cfDNA BCT (2 h); R^2^ = 0.876 for K_2_EDTA (2 h) vs cfDNA BCT (3 d); R^2^ = 0.962 for cfDNA BCT (2 h) vs cfDNA BCT (3 d). Robust test results were also obtained even for samples with low mutant molecule counts for which sampling variability caused by rare event statistics may limit the meaningfulness of the test results. Overall, the data suggest equivalent performance of cfDNA BCTs if clinical samples are stored for up to 3 d at RT compared to K_2_EDTA tubes stored for 2 h.

### Extreme storage temperatures lead to increased DNA release in cfDNA BCTs and lower plasma volumes

cfDNA BCTs may well be exposed to extreme temperatures or temperature changes during the shipping process to a testing laboratory. To evaluate the effect of temperatures outside of the 6–37°C range recommended by the manufacturer, blood from healthy donors (n = 6) collected in cfDNA BCTs was stored at 4°C, RT and 40°C for up to 5 days before plasma preparation (Fig 1, study cohort IV).

**Fig 6:**
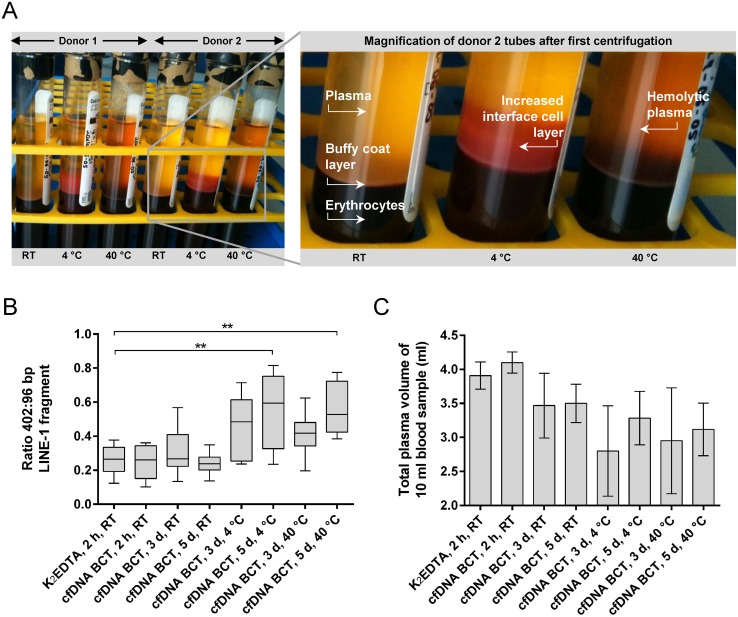
Effect of extreme storage temperatures on plasma separation and DNA yield in study cohort IV. (A) Representative image of cfDNA BCTs centrifuged after 3 days of storage at RT, 4°C and 40°C. RT storage resulted in expected plasma separation with defined buffy coat layer and clear yellow plasma fraction. Extreme temperature conditions resulted in an expanded cellular interface layer or hemolytic plasma at 4°C or 40°C, respectively. (B) Effect of extreme temperatures on genomic DNA release (402 bp LINE-1 qPCR fragment). Statistically significant differences between K_2_EDTA and cfDNA BCT storage conditions determined by one-way ANOVA are marked with ** (p ≤ 0.01). Shown are box plots with 1.5 x IQR applied to create whiskers. (C) Obtained mean plasma volume with SD for indicated storage conditions.

Visual differences between cfDNA BCTs stored at RT and at extreme temperatures were apparent already after the first centrifugation step during plasma preparation (Fig 6A). All RT storage conditions resulted in a clear plasma fraction with a defined buffy coat layer, whereas samples stored for 3 and 5 days at 4°C showed an expanded cellular interface layer above the buffy coat affecting 20–50% of the plasma fraction. Darker hemolytic plasma was visible for all samples stored at 40°C for a prolonged time. The visual differences in both high and low temperature conditions were accompanied by a time-dependent increase of long DNA fragments as illustrated by the elevated 402:96 bp ratios in Fig 6B. Storage at 4°C and 40°C for 5 days resulted in average 402:96 bp ratios between 0.5 and 0.6 and an overall larger variability between replicates. In contrast, the average 402:96 bp ratio for all RT conditions remained stable between 0.2 and 0.3, further confirming the observed cell-stabilizing effect at RT for up to 5 days. In addition to the increased DNA release, the average plasma volume was decreased by about 1 ml for samples stored at extreme temperatures compared to the control condition.

The results indicate that exposure to extreme temperatures outside of the recommended range of 6–37°C needs to be avoided for cfDNA BCTs in order to prevent dilution of potential mutant cfDNA molecules with wild-type genomic DNA released from WBCs.

### Maintaining a defined temperature range limits DNA release in cfDNA BCTs

The results obtained demonstrate that DNA release from WBCs in cfDNA BCT samples is time- and temperature-dependent. Thus, the following additional experiment was performed to quantify genomic DNA release and plasma volumes in samples collected from healthy donors (n = 8) and stored for 3 days at different temperatures within the recommended 6–37°C range (Fig 1, study cohort V).

**Fig 7:**
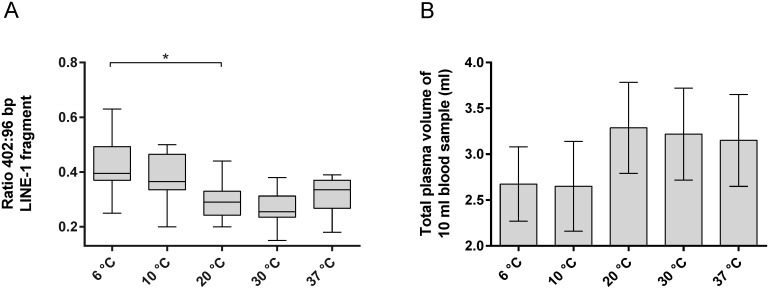
Genomic DNA release and obtained plasma volume after 3 days of storage within temperature range recommended by the manufacturer (study cohort V). Blood collected in cfDNA BCTs (n = 8) was stored for 3 days at the indicated temperature and subsequently analyzed using the genomic DNA release assay based on the 402:96 bp LINE-1 ratio (A). Shown are box plots with 1.5 x IQR applied to create whiskers. Statistically significant differences from the reference condition (20°C) were determined by one-way ANOVA and are marked with an * (p ≤ 0.05). (B) Obtained mean plasma volume with SD for indicated storage conditions.

Fig 7A demonstrates that 3 days of blood storage at lower temperatures contributes to genomic DNA release compared to the control condition (storage at 20°C). In particular, the 6°C condition exhibited a significantly increased median 402:96 bp ratio above 0.4 (p = 0.019). Elevated 402:96 bp ratios were associated with the presence of an expanded cell interface layer between the plasma and the buffy coat for samples stored at 6°C and 10°C and also correlated with a reduced plasma volume obtained from these samples (Fig 7B). Neither the presence of an interface layer nor hemolytic plasma was observed for blood stored between 20°C and 37°C, resulting in 402:96 bp ratios and recoverable plasma volumes within the expected range for cfDNA BCTs stored for 3 days. Taken together, these results demonstrate the importance of maintaining temperatures above 10°C and up to 37°C during the shipping process to ensure uncompromised plasma quality in cfDNA BCTs.

## Discussion

The results reported in the present study demonstrate that the pre-analytical workflow from blood collection to plasma preparation for liquid biopsy testing can be simplified by using cfDNA BCTs compared to standard K_2_EDTA blood collection tubes. It was confirmed that cfDNA and the nucleated cell population in blood samples collected in cfDNA BCTs are stabilized at room temperature, thus eliminating the need for on-site plasma processing and allowing standard room temperature shipping to the testing laboratory. Furthermore, it was shown that the sequence integrity of the cfDNA itself is not affected by the simplified workflow. Specifically, the cfDNA is still amplifiable and does not contain measurable DNA damage which enables the detection of low level mutations. Overall, cfDNA BCTs seem to be well suited for liquid biopsy applications in oncology.

Our results are consistent with others demonstrating that cfDNA BCTs can be utilized for prenatal diagnostics and mutation testing in oncology [5,11,15,21,29–31]. However, most of these studies did not use techniques sensitive enough to assess the mutation background in wild-type samples and detect low levels of mutant DNA molecules in a large background of normal DNA. Moreover, one of the distinguishing features of our study lies in the comprehensive evaluation of temperature effects rather than simply determining the usability under room temperature conditions. A detailed comparison of past findings with our observations is provided as supplementary information (S2 Table).

The outcome of our study suggests that the cfDNA BCT preservative stabilizes cfDNA for up to 5 days at RT and prevents the release of genomic DNA from blood cells confirming the results of other studies [11,21,30,31]. However, whether cfDNA levels are maintained over longer time periods with agitation, like during shipping, was previously addressed only for a maximum time of 48 hours [30,32]. Since a three day shipping process to the testing laboratory is assumed to be the standard situation encountered in clinical testing [21], we examined permanent agitation for up to 3 days. During these conditions no adverse effects were identified. It needs to be noted that agitation does not entirely reflect all movements that may occur during shipment. However, previous studies with fetal cfDNA confirmed no impact of shipment on total cfDNA quantity [31,32]. Whether the cfDNA integrity is maintained longer than 5 days without agitation or longer than 3 days with agitation was not determined.

To our surprise, our results suggest that storage temperatures have a much larger influence on the cfDNA population than initially assumed. We observed an effect on genomic DNA levels as well as plasma volumes at temperatures just a few degrees outside of the manufacturer’s specifications. For example, a 3–5 day storage at 4°C or 40°C resulted in up to 10-fold increase of larger normal genomic DNA fragments compared to a 2 h storage in standard K_2_EDTA tubes. A similar effect was described by Hidestrand *et al*. for prenatal applications when shipping blood on frozen gel packs [32]. Wong *et al*. demonstrated a slight but insignificant increase in cfDNA for samples stored in cfDNA BCTs at 4°C for 24 h, whereas storage at 23°C, 37°C and 40°C resulted in a significantly increased cfDNA concentration and a consequently reduced fetal DNA fraction [21]. In our study, both low and high storage temperatures resulted in elevated genomic DNA levels. For high quality plasma, long DNA fragments only represent 20% of the cfDNA (402:96 bp ratio of 0.2) as demonstrated by this study and others [33]. In contrast, storage of blood in cfDNA BCTs under suboptimal conditions resulted in low quality plasma with more than 60% of long wild-type DNA (402:96 bp ratio of 0.6). This change constitutes a 2-fold dilution of the ctDNA concentration. Low quality plasma will therefore affect the testing performance around the assay cut-off. Sherwood *et al*. demonstrated this effect for a qPCR-based test which failed to detect ctDNA in previously confirmed mutant samples if blood of the same donors was stored for 72 h in K_2_EDTA tubes [5]. With liquid biopsies being considered for monitoring disease burden or response to treatment, constant plasma quality will be of utmost importance to ensure reliable quantitative longitudinal measurements.

In addition to the above observations, the plasma color as well as the interface between plasma and red blood cells changed dramatically in extreme temperature conditions. Also the obtained plasma volume dropped on average from about 4 ml in the reference condition to about 3 ml in the extreme temperature conditions. At temperatures below RT (4–10°C), the volume reduction is mainly caused by the appearance of an expanded interface layer. Most importantly, our results indicate that prolonged storage even within the temperature range recommended by the manufacturer at 6–10°C resulted in the above described observations. Since these temperatures have an impact on the plasma quality, it is recommended to implement a temperature-controlled shipping process. Furthermore, a visual inspection of the blood collection tubes for abnormalities during the plasma preparation itself should be considered as a necessary quality control step to ensure that low quality plasma does not enter the testing process. These recommendations should become part of a standardized pre-analytical guideline to be introduced for liquid biopsy testing. Such standards should include methodological information and protocols about the blood collection, shipping, plasma preparation, and downstream DNA extraction, thus allowing for validation across analytical platforms and laboratories.

Our study also examined the effect of cfDNA BCTs on the mutation detection process which is crucial for liquid biopsy testing. This was of high interest to us, especially since it is assumed that cell stabilization reagents directly or indirectly cause DNA damage which may result in false positive mutation testing results [24]. Particularly critical are cell preservative-driven nucleotide changes such as cytosine-to-thymine conversions [25]. In this study, we did not detect an effect of the cfDNA BCT preservative on the mutation background of wild-type donor samples analyzed by BEAMing and Safe-SeqS after prolonged storage at RT. We were also able to show that the detection of low frequency spiked mutations was not impaired in samples stored in cfDNA BCTs. To our best knowledge, this is the first study to employ highly sensitive mutation profiling techniques to analyze a potential impact of cfDNA BCTs on the mutation background in healthy donor blood samples.

Lastly, applying mutation testing by BEAMing, we were able to demonstrate that mutant DNA fragments released from colorectal tumors can be found in the circulation at highly comparable detection rates and frequencies in cfDNA BCTs and standard K_2_EDTA tubes, even at very low MAFs. According to the common release mechanisms of cfDNA from tumors, it is anticipated that similar degrees of concordance will be achievable for other tumor types.

The cfDNA BCTs evaluated in this study can be considered a game changer for the broad adaption of liquid biopsy testing. As such, they have advantages and limitations. The advantage of the use of cfDNA BCTs over standard K_2_EDTA plasma tubes lies in the former device to preserve the integrity and compositions of the cfDNA population as it is present *in vivo* at the time of blood collection until the start of plasma processing in the testing laboratory. One limitation is that the cfDNA BCTs are not suitable for the parallel analysis of other biomarkers such as proteins, DNA methylation [34] and circulating RNA. Another limitation of these tubes is the need for a modification to the DNA extraction procedure. Specifically, an extended heating step is required to reverse chemical fixations [30]. This means that any DNA extraction procedure without an extended heating step would require further testing to examine its compatibility with cfDNA BCTs. Lastly, as described above, a temperature-controlled shipping process is necessary to maintain stability of the WBCs for storage and shipping of blood collected in cfDNA BCTs.

In summary, we have presented a comprehensive evaluation of cfDNA BCTs for liquid biopsy testing. We expect that theses tubes can be applied for any cancer type and liquid biopsy application using BEAMing and Plasma Safe-Sequencing (Safe-SeqS). Indeed, the CE-IVD version of the cfDNA BCTs is now recommended as the preferred collection device for the CE-IVD OncoBEAM RAS CRC kit, a liquid biopsy test for *RAS* mutation profiling in colorectal cancer [28]. Even if these tubes have some limitations with respect to the temperature ranges as well as biomarker compatibility, liquid biopsy testing can now be performed for patients who previously missed easy access to this type of molecular mutation profiling.

## Supporting Information

S1 Fig Total plasma volume obtained from healthy donor samples collected in K_2_EDTA and cfDNA BCTs (study cohort I). Total plasma volume was assessed for cfDNA from blood samples stored at RT in K_2_EDTA tubes and cfDNA BCTs (healthy donors, n = 60). Plasma was prepared after indicated storage conditions. Shown are box plots with 1.5 x IQR applied to create whiskers and outliers.
(TIFF)

S2 Fig Representation of mutant fractions in cfDNA BCTs relative to matched K_2_EDTA values (study cohort II). MAF ratios between cfDNA BCT and matched K_2_EDTA reference values were calculated for all spiked donor samples and mutations (*PIK3CA* c.3140A>G spike (0.1%), *EGFR* c.2369C>T spike (0.5%), *KRAS* c.34G>A spike (1%)). Shown are all resulting relative values with mean (horizontal line) ± SD.
(TIFF)

S1 Table Mutation analysis results from CRC samples collected in K_2_EDTA and cfDNA BCTs (study cohort III). DNA extracted from 2 ml plasma was quantified using the 96 bp LINE-1 qPCR assay. BEAMing was used to test for *RAS* mutations, and the number of mutant molecules was determined by multiplying the mutant fraction by the respective DNA amount of the sample.
(DOCX)

S2 Table Comparison of results obtained in this study with previously published findings. (DOCX)
