# Supplementary figures and images for: Performance of Streck cfDNA Blood Collection Tubes for Liquid Biopsy Testing
Source: PLoS One. 2016 Nov 10;11(11):e0166354. doi: 10.1371/journal.pone.0166354 (PMC5104415; doi:10.1371/journal.pone.0166354)

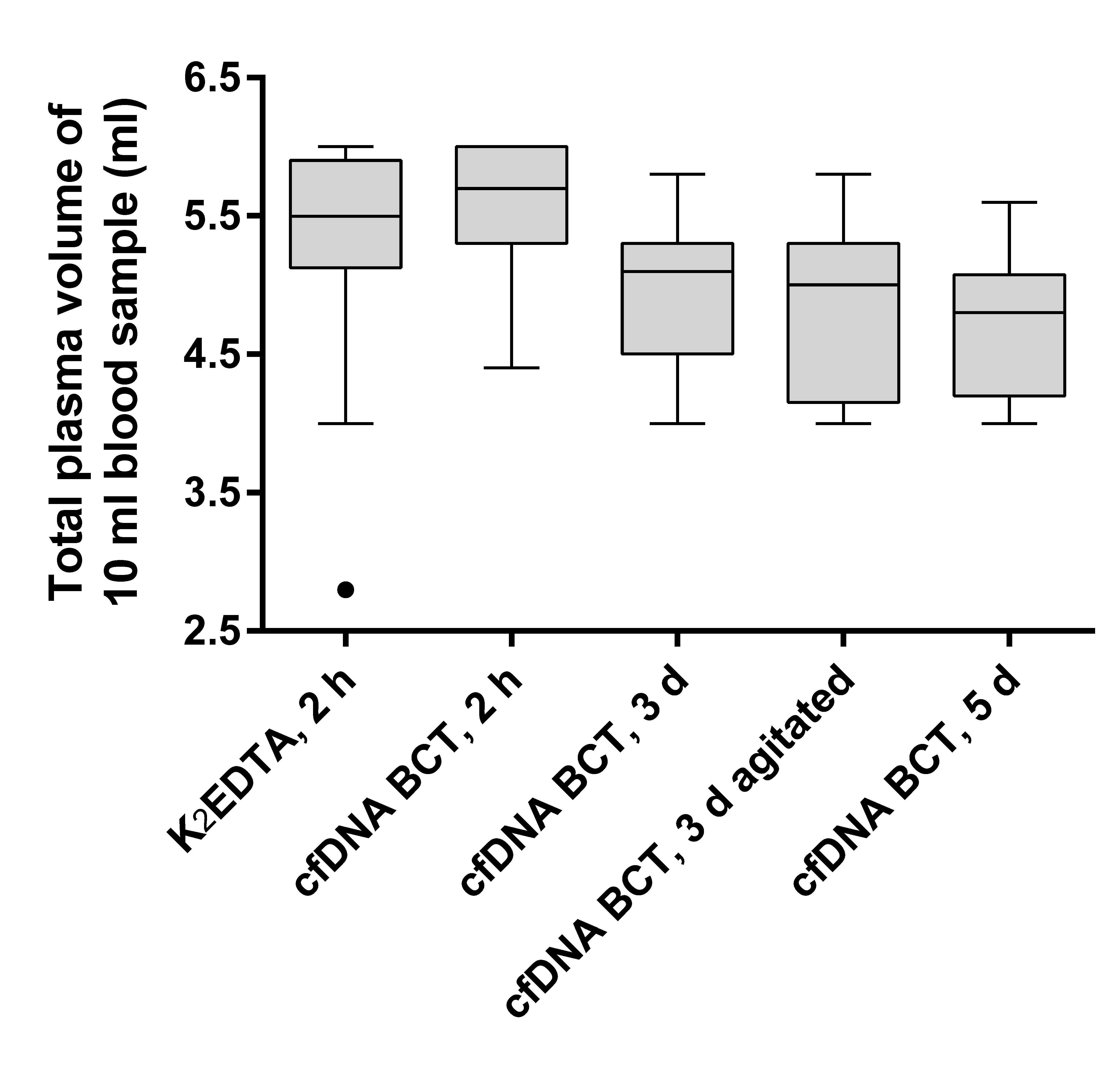

Supplement: S1 Fig — Total plasma volume was assessed for cfDNA from blood samples stored at RT in K2EDTA tubes and cfDNA BCTs (healthy donors, n = 60). Plasma was prepared after indicated storage conditions. Shown are box plots with 1.5 x IQR applied to create whiskers and outliers. (TIFF) [file pone.0166354.s001.tiff]

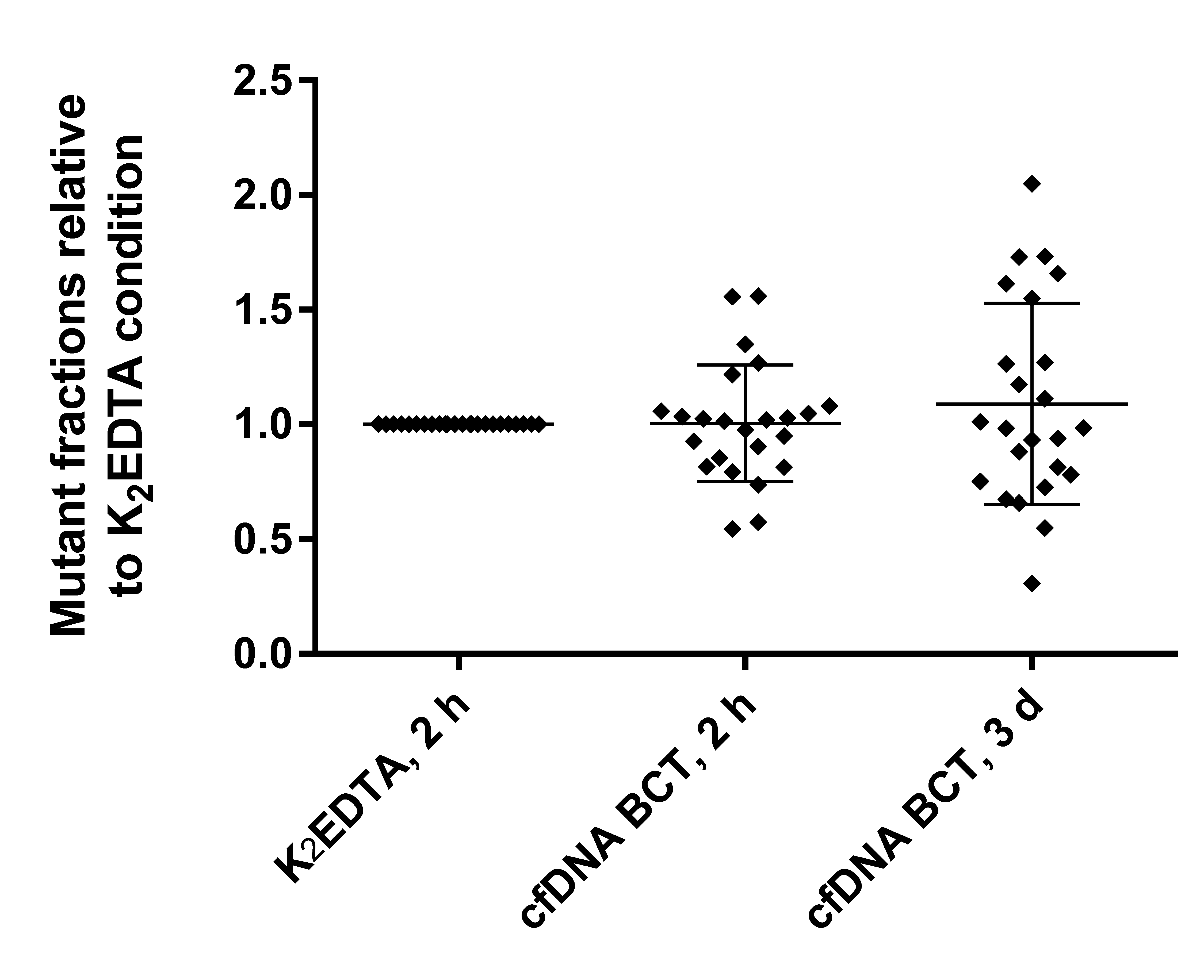

Supplement: S2 Fig — MAF ratios between cfDNA BCT and matched K2EDTA reference values were calculated for all spiked donor samples and mutations (PIK3CA c.3140A>G spike (0.1%), EGFR c.2369C>T spike (0.5%), KRAS c.34G>A spike (1%)). Shown are all resulting relative values with mean (horizontal line) ± SD. (TIFF) [file pone.0166354.s002.tiff]
