# Supplementary material for: Performance of Streck cfDNA Blood Collection Tubes for Liquid Biopsy Testing
Source: PLoS One. 2016 Nov 10;11(11):e0166354. doi: 10.1371/journal.pone.0166354 (PMC5104415; doi:10.1371/journal.pone.0166354)
Supplement: S1 Table — DNA extracted from 2 ml plasma was quantified using the 96 bp LINE-1 qPCR assay. BEAMing was used to test for RAS mutations, and the number of mutant molecules was determined by multiplying the mutant fraction by the respective DNA amount of the sample. (DOCX) [file pone.0166354.s003.docx]

**S1 Table. Mutation analysis results from CRC samples collected in K_2_EDTA and cfDNA BCTs (study cohort III).** DNA extracted from 2 ml plasma was quantified using the 96 bp LINE-1 qPCR assay. BEAMing was used to test for *RAS* mutations, and the number of mutant molecules was determined by multiplying the mutant fraction by the respective DNA amount of the sample.

| **Sample ID** | **Stage** | **Mutation** | **Mutant fraction (%)** | **Total DNA Amount (GE)** | **Mutant molecules** |
| --- | --- | --- | --- | --- | --- |
| Donor 2, K_2_EDTA, 2 h, RT | IVB | KRAS_g38a | 32.83 | 117553 | 38593 |
| Donor 2, cfDNA BCT, 2 h, RT |  |  | 41.63 | 93975 | 39119 |
| Donor 2, cfDNA BCT, 3 d, RT |  |  | 40.84 | 94172 | 38457 |
| Donor 3, K_2_EDTA, 2 h, RT | IVA | KRAS_g34t | 27.86 | 70947 | 19768 |
| Donor 3, cfDNA BCT, 2 h, RT |  |  | 26,24 | 45306 | 11887 |
| Donor 3, cfDNA BCT, 3 d, RT |  |  | 16.87 | 58550 | 9877 |
| Donor 5, K_2_EDTA, 2 h, RT | IIA | KRAS_g38a | 0.02 | 21197 | 4 |
| Donor 5, cfDNA BCT, 2 h, RT |  |  | 0.11 | 18873 | 21 |
| Donor 5, cfDNA BCT, 3 d, RT |  |  | 0.05 | 21505 | 11 |
| Donor 7, K_2_EDTA, 2 h, RT | IV | KRAS_g35a | 24.36 | 518814 | 126362 |
| Donor 7, cfDNA BCT, 2 h, RT |  |  | 27.25 | 554853 | 151175 |
| Donor 7, cfDNA BCT, 3 d, RT |  |  | 22.70 | 501963 | 113966 |
| Donor 7, K_2_EDTA, 2 h, RT | IV | NRAS_g34a | 0.03 | 518814 | 151 |
| Donor 7, cfDNA BCT, 2 h, RT |  |  | 0.03 | 554853 | 144 |
| Donor 7, cfDNA BCT, 3 d, RT |  |  | 0.04 | 501963 | 216 |
| Donor 10, K_2_EDTA, 2 h, RT | IIIB | KRAS_a183t | 0.16 | 14514 | 24 |
| Donor 10, cfDNA BCT, 2 h, RT |  |  | 0.10 | 14883 | 15 |
| Donor 10, cfDNA BCT, 3 d, RT |  |  | 0.17 | 23001 | 39 |
| Donor 18, K_2_EDTA, 2 h, RT | IVB | KRAS_g35t | 3.88 | 28816 | 1119 |
| Donor 18, cfDNA BCT, 2 h, RT |  |  | 2.92 | 21746 | 636 |
| Donor 18, cfDNA BCT, 3 d, RT |  |  | 2.31 | 20766 | 479 |
