# Supplementary material for: Performance of Streck cfDNA Blood Collection Tubes for Liquid Biopsy Testing
Source: PLoS One. 2016 Nov 10;11(11):e0166354. doi: 10.1371/journal.pone.0166354 (PMC5104415; doi:10.1371/journal.pone.0166354)
Supplement: S2 Table — (DOCX) [file pone.0166354.s004.docx]

**S2 Table. Comparison of results obtained in this study with previously published findings.**

| **Topic** | **Observations Medina Diaz *et al.*** | **Observations in the literature** | **Ref.** |
| --- | --- | --- | --- |
| **Choice of blood collection tube** | - Performance of Streck cfDNA BCTs is comparable to K_2_EDTA and suitable for downstream liquid biopsy testing by BEAMing and Safe-SeqS | - Streck cfDNA BCT frequently applied for prenatal testing (K_3_EDTA collection tubes or cfDNA BCT recommended) - Use of cfDNA BCTs advantageous if rapid sample processing not possible (NSCLC; Therascreen) - Use of cfDNA BCTs could be of benefit for blood specimen collections in clinical trials (metastatic breast cancer: Droplet digital PCR) - cfDNA BCT, CellSave, and K_3_EDTA tube effectively stabilize ctDNA and gDNA. cfDNA BCTs provides extended stability if kept at RT (metastatic breast cancer; Droplet digital PCR) | [1,2]  [3]  [4]  [5] |
| **Storage time influence (cfDNA and WBC stabilization)** | - Stable cfDNA yields and no detectable genomic DNA release from WBCs for up to 5 d at RT in cfDNA BCTs compared to 2 h RT storage in K_2_ETDA or cfDNA BCTs. | - Stable cfDNA and gDNA yields in cfDNA BCTs up to 7 d at RT, whereas gDNA increased 8-fold at day 14 compared to day one - Stable cfDNA and gDNA yields in cfDNA BCTs up to 7 d at RT - Stable cfDNA and gDNA yields in cfDNA BCTs up to 48 h at RT | [6]  [4]  [5] |
| **Storage temperature influence (cfDNA and WBC stabilization)** | - RT storage in cfDNA BCTs results in stable cfDNA yields and no detectable genomic DNA release for up to 5 d (compared to 2 h RT storage in K_2_ETDA or cfDNA BCTs) - 3 - 5 d storage in cfDNA BCTs at 4 °C or 40 °C shows up to 10-fold increase of genomic DNA fragments compared to a 2 h storage in standard K_2_EDTA tubes - 6 °C storage in cfDNA BCTs for 3 d shows a slight but significant increase in gDNA/cfDNA ratio compared to 3 d RT storage | - No difference in gDNA/cfDNA ratio for 4 °C storage compared to RT storage (6 h, K_2_EDTA tubes) - No difference in cfDNA concentration for 24 h storage at 4 °C compared to 8 h storage at RT storage (K_3_EDTA tubes) - No difference in gDNA/cfDNA ratio for 8 h storage at 4 °C compared to RT storage in K_3_EDTA tubes. cfDNA BCTs beneficial if storage at RT > 8 h (prenatal testing) - cfDNA concentration does not vary significantly within 4-6 h following venipuncture at RT or at 4 °C (EDTA tube) - Blood samples collected in cfDNA BCTs should not be stored in a refrigerator and/or freezer since this induces cell lysis (unpublished observations) - cfDNA BCTs effectively stabilize ctDNA and gDNA for 6 h at 4 °C and RT. 2 out of 6 patients showed an increase of wild-type DNA at 48 h in cfDNA BCTs stored on ice, but not at RT | [7]  [8]  [9]  [1]  [4]  [5] |
| **Agitation influence (cfDNA and WBC stabilization)** | - 3 d permanent agitation of cfDNA BCTs does not alter cfDNA yields or genomic DNA release | - Agitation of blood samples for 3 h at RT causes a slight increase of cfDNA in EDTA tubes - No significant change in cfDNA was detected in plasma if cfDNA BCT samples were shipped overnight at RT; Significant decrease in fetal DNA fraction was found in EDTA samples and cfDNA BCTs shipped overnight at 4 °C (prenatal testing) - Shaking and shipment of blood in K_3_EDTA tubes shows a significant increases in gDNA, whereas no change is seen in cfDNA BCTs (RT, up to 24 h or RT 4 d shipment; prenatal field) | [1]  [10]  [6] |
| **Optical appearance of plasma fraction** | - Abnormal visual appearance of the plasma fraction during preparation of plasma from cfDNA BCT tubes shows strong correlation to plasma quality (broad interface cell layers, hemolytic plasma; increasing gDNA levels); visual appearance of plasma during preparation should be considered as a necessary quality control step | - n/a | n/a |
| **cfDNA amplifiability** | - cfDNA BCTs do not impair PCR amplifiability of cfDNA | - Reagent used in cfDNA BCTs has no effect on DNA amplification for storage up to 14 d at RT, whereas formaldehyde and glutaraldehyde treated DNA shows a time dependent decrease in DNA amplification indicating DNA damage - cfDNA BCTs containing reagent does not affect downstream molecular analysis by PCR (Therascreen & Digital droplet PCR) - cfDNA BCTs are not suitable for subsequent analysis of mSEPT9 with the Epi proColon 2.0 CE Early Detection Assay (the assay is a duplex PCR determining methylation of SEPT9) | [11]  [3–5]  [12] |
| **Mutation background in wild-type samples** | - No detectable effect of the cfDNA BCT preservative on the mutation background level of wild-type donor samples (BEAMing: 5 KRAS point mutations; Safe-SeqS: 5 c-KIT amplicons) | - n/a | n/a |
| **Detectability of mutant allele frequencies** | - Mutational load in CRC cancer samples was highly comparable between K_2_EDTA tubes stored for 2 h and Streck cfDNA BCTs stored for up to 3 d at RT - The detection of low frequency spiked mutations (0.1%, 0.5%, 1%) is not impaired in samples stored in cfDNA BCTs compared to K_2_EDTA tubes | - Successful ctDNA recovery for mutation detection in NSCLC with cfDNA BCTs vs EDTA (Therascreen) - Successful ctDNA recovery for mutation detection in metastatic breast cancer patients with cfDNA BCTs after 48 h or 7 d RT storage (Droplet digital PCR) | [3]  [4,5] |
| **Plasma volumes** | - Dropped slightly over 5 d RT storage in cfDNA BCTs - Dropped >1 ml and was highly variable at 4 °C and 40° C storage for 3 d & 5 d in cfDNA BCTs | - n/a | n/a |

**References**

1. El Messaoudi S, Rolet F, Mouliere F, Thierry AR. Circulating cell free DNA: Preanalytical considerations. Clin Chim Acta. 2013;424: 222–30. doi:10.1016/j.cca.2013.05.022

2. Wang Q, Cai Y, Brady P, Vermeesch JR. Real-time PCR evaluation of cell-free DNA subjected to various storage and shipping conditions. Genet Mol Res. 2015;14: 12797–12804. doi:10.4238/2015.October.19.23

3. Sherwood JL, Corcoran C, Brown H, Sharpe AD, Musilova M, Kohlmann A. Optimised Pre-Analytical Methods Improve KRAS Mutation Detection in Circulating Tumour DNA (ctDNA) from Patients with Non-Small Cell Lung Cancer (NSCLC). PLoS One. 2016;11: e0150197. doi:10.1371/journal.pone.0150197

4. Toro PV, Erlanger B, Beaver J a, Cochran RL, VanDenBerg D a, Yakim E, et al. Comparison of cell stabilizing blood collection tubes for circulating plasma tumor DNA. Clin Biochem. Elsevier B.V.; 2015;48: 993–8. doi:10.1016/j.clinbiochem.2015.07.097

5. Kang Q, Henry NL, Paoletti C, Jiang H, Vats P, Chinnaiyan AM, et al. Comparative Analysis of Circulating Tumor DNA Stability In K3EDTA, Streck and CellSave Blood Collection Tubes. Clin Biochem. Elsevier B.V.; 2016; doi:10.1016/j.clinbiochem.2016.03.012

6. Norton SE, Luna KK, Lechner JM, Qin J, Fernando MR. A New Blood Collection Device Minimizes Cellular DNA Release During Sample Storage and Shipping When Compared to a Standard Device. J Clin Lab Anal. 2013;27: 305–311. doi:10.1002/jcla.21603

7. Chan KCA, Yeung S-W, Lui W-B, Rainer TH, Lo YMD. Effects of preanalytical factors on the molecular size of cell-free DNA in blood. Clin Chem. 2005;51: 781–4. doi:10.1373/clinchem.2004.046219

8. Jung M, Klotzek S, Lewandowski M, Fleischhacker M, Jung K. Changes in concentration of DNA in serum and plasma during storage of blood samples [5]. Clin Chem. 2003;49: 1028–1029. doi:10.1373/49.6.1028

9. Barrett AN, Zimmermann BG, Wang D, Holloway A, Chitty LS. Implementing prenatal diagnosis based on cell-free fetal DNA: accurate identification of factors affecting fetal DNA yield. Novelli G, editor. PLoS One. Public Library of Science; 2011;6: e25202. doi:10.1371/journal.pone.0025202

10. Hidestrand M, Stokowski R, Song K, Oliphant A, Deavers J, Goetsch M, et al. Influence of Temperature during Transportation on Cell-Free DNA Analysis. Fetal Diagn Ther. 2012;31: 122–128. doi:10.1159/000335020

11. Das K, Fernando MR, Basiaga S, Wigginton SM, Williams T. Effects of a novel cell stabilizing reagent on DNA amplification by PCR as compared to traditional stabilizing reagents. Acta Histochem. Elsevier GmbH.; 2013; doi:10.1016/j.acthis.2013.05.002

12. Distler J, Tetzner R, Weiss G, König T, Schlegel A, Bagrowski M. Evaluation of different blood collection tubes and blood storage conditions for the preservation and stability of cell-free circulating DNA for the analysis of the methylated mSEPT9 colorectal cancer screening marker. P 209. Berlin; 2015. p. 69. Available: http://www.cnaps-congress.com/_static/media/CNAPS_2015_Programm_A4_PP_web.pdf
